# A Review on Expression, Pathological Roles, and Inhibition of TMPRSS2, the Serine Protease Responsible for SARS-CoV-2 Spike Protein Activation

**DOI:** 10.1155/2021/2706789

**Published:** 2021-07-24

**Authors:** Jyotirmoy Sarker, Pritha Das, Sabarni Sarker, Apurba Kumar Roy, A. Z. M. Ruhul Momen

**Affiliations:** ^1^Department of Pharmacy, Jagannath University, Dhaka 1100, Bangladesh; ^2^Department of Pharmacy Systems, Outcomes and Policy, University of Illinois at Chicago, Chicago, IL 60607, USA; ^3^Independent Author, Dhaka 1207, Bangladesh; ^4^Department of Genetic Engineering and Biotechnology, University of Rajshahi, Rajshahi 6205, Bangladesh

## Abstract

SARS-CoV-2, the coronavirus responsible for the COVID-19 pandemic, uses the host cell membrane receptor angiotensin-converting enzyme 2 (ACE2) for anchoring its spike protein, and the subsequent membrane fusion process is facilitated by host membrane proteases. Recent studies have shown that transmembrane serine protease 2 (TMPRSS2), a protease known for similar role in previous coronavirus infections, severe acute respiratory syndrome (SARS), and Middle East respiratory syndrome (MERS), is responsible for the proteolytic cleavage of the SARS-CoV-2 spike protein, enabling host cell fusion of the virus. TMPRSS2 is known to be expressed in the epithelial cells of different sites including gastrointestinal, respiratory, and genitourinary system. The infection site of the SARS-CoV-2 correlates with the coexpression sites of ACE2 and TMPRSS2. Besides, age-, sex-, and comorbidity-associated variation in infection rate correlates with the expression rate of TMPRSS2 in those groups. These findings provide valid reasons for the assumption that inhibiting TMPRSS2 can have a beneficial effect in reducing the cellular entry of the virus, ultimately affecting the infection rate and case severity. Several drug development studies are going on to develop potential inhibitors of the protease, using both conventional and computational approaches. Complete understanding of the biological roles of TMPRSS2 is necessary before such therapies are applied.

## 1. Background

The novel coronavirus disease or COVID-19 emerged in Wuhan province of China in late 2019 and rapidly spread to almost every country and territory around the world within months. According to the interactive online tracker developed by Center for Systems Science and Engineering at Johns Hopkins University, the global number of confirmed cases till 30 May 2021 reached nearly 170,000,000, causing more than 3,500,000 deaths [1]. Understanding the sequence of events that lead to the infection of human cells by the virus can facilitate the process of investigating potential intervention options. SARS-CoV-2, the virus responsible for COVID-19, belongs to the Coronaviridae family [2]. Members of this family are single-stranded RNA virus with glycoprotein spikes attached to envelope [2]. These spikes are responsible for anchoring the virus to the ACE2 receptor in the host cell surface [2]. Studies have found that the fusion between virus and host cell membrane is facilitated by some host cell membrane protease enzymes, which cleaves the spike protein of the virus envelope and enables the fusion process [2]. TMPRSS2, a serine transmembrane protease, has been predicted to play this role for SARS-CoV-2 [2]. This protease has been known for long for its association with prostate cancer and some other viral infections, including influenza, SARS, and MERS. As a result, TMPRSS2 has been in the center of attention of drug developers in recent months, leading to several studies for finding a potential solution to reduce TMPRSS2 expression or inhibit its activity in the host cell membranes, so that the cellular entry of SARS-CoV-2 can be reduced. Here, the role of TMPRSS2 protease in SARS-CoV-2 infection and its expression sites and pathological roles are briefly discussed. Furthermore, some ongoing approaches to inhibit the protease are highlighted.

## 2. Host Cell Entry of SARS-CoV-2 and the Role of TMPRSS2

**Figure 1:**
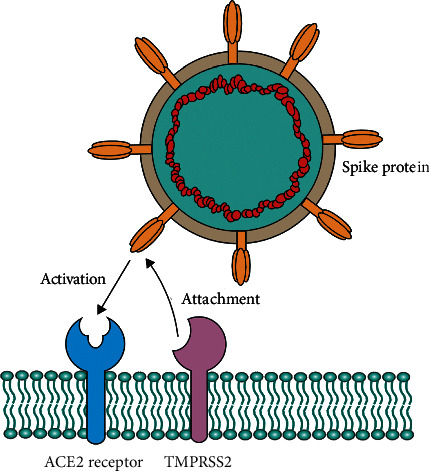
TMPRSS2 plays an important role in activation and conformational change of SARS-CoV-2 spike protein, which leads to the ACE2 receptor binding of the virus.

**Figure 2:**
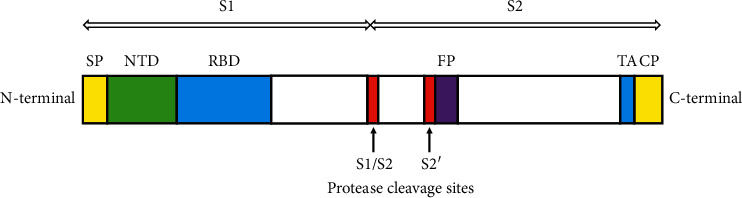
Schematic diagram of the domain structure of SARS-CoV-2 spike (S) protein. SP = signal peptide, NTD = N-terminal domain, RBD = receptor binding domain, FP = fusion peptide, TA = transmembrane anchor, and CP = cytoplasmic domain [11].

SARS-CoV-2 is a positive-sense single-stranded RNA virus of the *Betacoronavirus* genus of the Coronaviridae family [3, 4]. This virus is constructed of four major structural proteins along with sixteen nonstructural and five to eight accessory proteins [4]. The transmembrane spike protein (S), a 150 kDa structural glycoprotein, is associated with host cell anchoring of the virus [4–6]. The S protein of SARS coronavirus, which was responsible for the 2002-2003 pandemic, interacts with the angiotensin-converting enzyme 2 (ACE2) in host cell surface [7]. The S protein of SARS-CoV and SARS-CoV-2 has 76% sequence similarity [8]. Due to this high level of similarity, it was assumed that SARS-CoV-2 also interacts with the ACE2 receptor in host cell for cellular entry (Figure 1) [8, 9]. Later, studies conducted by Zhou et al. and Wan et al. showed that SARS-CoV-2 can use the ACE2 receptor for cellular entry [3, 9]. Specific domains of the spike protein are responsible for receptor attachment, protease processing, and cell membrane fusion [10]. The extracellular domain, one of three domains of the S protein, has two functional subunits, S1 and S2 [8, 11]. The amino terminal S1 subunit is responsible for bindings with host cell receptor and the carboxy terminal S2 for membrane fusion [4, 8]. S1 subunit is further divided into N-terminal domain (NTD) and receptor binding domain (RBD) [4]. The virus attaches with the host cell receptor ACE2 using the RBD [5]. Different domains of the S protein are shown in Figure 2.

The fusion of SARS-CoV-2 with host cell requires coordination between receptor binding and proteolysis of the spike protein (Figure 1) [8]. After spike protein cleavage by the host cell proteases, at the S1/S2 boundary and at another cleavage site of the S2 domain, S2′, the fusion peptide of the spike protein is released (Figure 2) [11]. Different host proteases are associated with the splitting of coronavirus spike proteins and subsequent cellular entry of different coronavirus, which eventually is a decisive factor for host and tissue specificity of the virus [10]. Also, there are some lines of evidence that the proteases take part in activating the host cell receptors for coronavirus attachment [10].

TMPRSS2, a type II transmembrane-bound serine protease constituted of 492 amino acids, is found to be involved in priming of S protein in SARS coronavirus [12]. In case of MERS virus spike protein, the cleavage at S1/S2 site is carried out by the proteolytic enzyme furin, and the subsequent S2′ cleavage is carried out by TMPRSS2 [13]. Hoffmann et al. confirmed the presence of similar multibasic S1/S2 cleavage site in SARS-CoV-2, which requires precleavage by furin before proteolytic activation of the fusion domain by TMPRSS2 [13].

This step is important for the fusion of virus and cellular membrane. In an earlier study, Hoffmann et al. found similar involvement of TMPRSS2 in the priming of S protein in SARS-CoV-2 and concluded that TMPRSS2 is essential for the cellular entry of SARS-CoV-2 [7]. The gene coding for this protease is 44 kB in length and has 14 exons [12, 14]. The different domains of TMPSSR2 are a type II transmembrane domain, a receptor class A domain, a scavenger receptor cysteine-rich domain, and a protease domain. The transmembrane domain has intracellular amino terminal and extracellular carboxy terminal, to which the protease domain is attached [15, 16]. For the enzyme to exert its proteolytic activity, autocleavage of the proteolytic domain and its subsequent secretion in the cell media are necessary [12].

## 3. Expression of TMPRSS2

### 3.1. Expression in Respiratory Tract

There is evidence of high level of TMPRSS2 expression in epithelial cells of different sites of the upper and lower respiratory system. Expression of TMPSSR2 in respiratory epithelial cells was mentioned in 2001 by Donaldson et al., when they found evidence of expression in the epithelial lining of the nose, trachea, and airways [17]. They predicted TMPRSS2 expression in type II pneumocytes of the pulmonary alveoli [17]. Later, Bertram et al. found that TMPRSS2 along with another protease is expressed in epithelial cells throughout the entire respiratory tract including lung, bronchus, larynx, trachea, nasal mucosa, and respiratory sinuses [18]. Evidence of expression was found in different cells of lung and bronchial branches, where the expression level in lung tissue was higher than that in bronchial epithelial cells [19]. Recent studies confirmed higher level of TMPRSS2 expression in type II of the lung alveolar epithelium cells than in type I cells [19, 20].

### 3.2. Expression in Gastrointestinal Tract

A northern blot analysis conducted by Paoloni-Giacobino et al. first predicted the expression of TMPSSR2 in the small intestine [12, 21]. Significant expression of TMPRSS2 was observed in epithelial and gland cells of the upper esophagus, but expression level was low in the stratified epithelial cells [22]. In stomach, the gene is highly expressed in the basal gland mucous cells, pit mucous cells, and chief cells [22]. TMPRSS2 expression was also observed in the epithelial cells of ileum and enterocytes of the colon [22]. Overall, the expression levels in upper esophagus, ileum, and colon enterocytes were higher than those in other cells [22]. From RNA-seq data, it was found that the gene was highly expressed in stomach, small intestine, and transverse colon, but the expression level was low in the sigmoid colon [22]. Burgueño et al. mentioned the significant expression of TMPRSS2 in human ileum and colon and the high level of expression in mouse duodenum [23]. Another recent *in vitro* study found that the human colorectal epithelial cell line Caco-2 can express TMPRSS2 [7].

### 3.3. Expression in Prostate

Lin et al. reported that the serine protease TMPRSS2 expression is much higher in prostate epithelium cells than in other human tissues [24]. Applying *in situ* hybridization technique, they found localized expression in the prostate basal cells but failed to observe any expression in the luminal secretory cells [24]. The same study also mentioned the androgen-dependent expression pattern of TMPRSS2 [24]. Later, Vaarala et al. found expression in luminal epithelial cells but no expression in basal cells [21]. Studies conducted afterwards supported the findings of Vaarala et al. that TMPRSS2 expression is found in the luminal cells of the prostate [15, 25]. Another group found that the exact expression of TMPRSS2 in prostate is from the apical surface of the luminal epithelial cells and this protease is also released in the prostasomes of the seminal fluid [26].

### 3.4. Expression in Ocular Tissue

Whether the cornea is an expression site of ACE2 and associated proteins has been of particular interest in recent times due to its possible implication in predicting the vulnerability of conjunctiva as an infection site for SARS-CoV-2. Zhang et al. found significant expression of TMPRSS2 in corneal epithelium, conjunctival epithelium, and lacrimal gland serous cells of mice with higher level of expression in conjunctiva than in cornea [27]. Later, another study on human conjunctiva tissue showed absence of TMPRSS2 expression [28]. Ma et al. found higher level of TMPRSS2 expression in mouse cornea than in conjunctiva [29].

### 3.5. Expression in Olfactory Epithelium

The olfactory epithelium of the nasal cavity is another location where this protease is expressed [30]. A study conducted in mouse models found that expression level is higher in the sustentacular cells of olfactory epithelia than in the receptor neurons [30]. The expression level of the protease increases with age [30]. It is predicted that TMPRSS2 is expressed in both neuronal and nonneuronal epithelial cells of the olfactory epithelium and the expression level is higher in comparison with the expression of ACE2 receptor protein in these sites [31]. Another RNA-seq study found expression only in subpopulation of the olfactory receptor neurons [31, 32]. Association between the expression patterns in these cells with reported cases of Anosmia is predicted.

## 4. TMPRSS2 Expression and Relation to SARS-CoV-2

### 4.1. Respiratory Tract

In case of SARS-CoV-2, researchers observed that the viral activation requires proteolytic action of TMPRSS2 and furin [33]. Laporte et al. claimed that the spike protein of the virus was adaptive to adjust the polymorphism of proteases of human airways, both TMPRSS2 and TMPRSS13 [34]. In some studies, the exact role of proteases and relation to ACE2 binding in nasopharyngeal was not linear [35]. However, in a case study, Rossi et al. observed that expression of proteases and ACE2 in nasopharyngeal region had a direct relation to the severity of the disease [36]. Some conditions such as obesity were observed to be responsible for the expression level of TMPRSS2, thus influencing the rate of the viral infection [37].

### 4.2. Gastrointestinal Tract

Expression of the protease TMPRSS2 is well documented in the intestinal tract [12]. In fact, DNA analysis revealed that the high expression of the protease in lung and intestine makes both organs vulnerable to SARS-CoV-2 [38]. Around a quarter of SARS-CoV-2 cases were marked by gastrointestinal (GI) tract infections [39]. Expression of the protease in various GI cells was one of the reasons behind this. For instance, intestinal enterocytes, especially those having well-defined brush borders, were found to be more susceptible to the virus due to higher expression of the protease [40–42]. Lee et al. experimented with an enterocyte cell line and reported the fact that enterocytic differentiation influenced viral propagation [42].

### 4.3. Genitourinary System

TMPRSS2 gene is expressed in male spermatogonia besides prostate cells [43]. This fact indicated that male reproductive system should be vulnerable to the SARS-CoV-2 infection. However, there is no direct evidence of infection being present in the semen of infected patients [44]. Nevertheless, scientists suggested that the several gonad diseases such as orchitis have a direct correlation with SARS-CoV-2 infection [45]. More possible alterations of gonad function due to the disease are debated and possible involvements of TMPRSS2 need to be sorted out [46].

### 4.4. Ocular and Olfactory System

In an early study in China, only 8 patients in 1000 showed ocular inflammation associated with SARS-CoV-2 [47]. However, relatively high expression of TMPRSS2 and ACE2 in conjunctiva suggests a possible transmission pathway in ocular tissue [48]. Another investigation reported that coexpression of ACE2, TMPRSS2, and mitochondrial genes could make cornea a potential and important contributor for the viral infection [49]. It seemed that, despite having a considerable concentration of proteases, SARS-CoV-2-associated conjunctivitis is rare while conjunctiva and cornea can be responsible for the transmission of the virus [50]. On the other hand, different types of cells express TMPRSS2 gene in the olfactory epithelium [30]. Researchers found that odor disturbance of SARS-CoV-2 patients is due to the presence of proteases and ACE2 receptors in nonneuronal olfactory cells [51].

### 4.5. Correlation between TMPRSS2 Expression Pattern and Disease Severity

Some known variables of COVID-19 predisposition and disease severity are correlated with TMPRSS2 expression pattern. It is assumed that age-, race-, or gender-specific variation of TMPRSS2 expression can explain the disease severity of SARS-CoV-2. In the case of pediatric patients, with the same level of viral loads, TMPRSS2, and ACE2 level as adults, they tend to express less severity of the disease. According to a recent study, this was due to the strong innate immune response of the children [52]. Another hypothesis was that children before puberty have lower levels of steroid hormones which are necessary for the upregulation of TMPRSS2 and thus are less susceptible to the severity of the disease [53].

It was observed that severity of COVID-19 is associated with the polymorphisms of ACE2 and TMPRSS2 expressing genes [54]. Studies showed that Asians showed higher expression of ACE2 gene and that might made them more vulnerable to COVID-19 than African and Caucasian races [55, 56]. As the expression of TMPRSS2 is regulated by androgen, it can have a potential role in the male predominance of the infection [24, 25, 57]. The absence of androgen in preadolescents can be a reason behind the low incidence in this age group [57].

There were early reports that smokers are more prone to serious forms of COVID-19 infection than nonsmokers, with more frequent need of ventilation and ICU support [58, 59]. Previous studies have shown that smoking increases androgen-to-estrogen ratio [57]. As TMPRSS2 is androgen-regulated, smoking increases TMPRSS2 expression level, which might eventually be responsible for the increased predisposition of smokers to SARS-CoV-2 infection. A recent systematic review by Hou et al. supports this assumption and states that smoking is independently related to increased mortality due to COVID-19 [60].

Chaklader et al. found upregulation of TMPRSS2 expression in the lung and oral epithelium tissue of smokers after analysing RNA sequencing data from The Cancer Genome Atlas [59]. Prostatic hypertrophy, a common problem of the elderly men, may have a role in increased TMPRSS2 expression, which may account for the increased severity of the infection in this age group [57]. In a recent case-control study, it was shown that TMPRSS2 expression along with ACE2 in the nasopharyngeal area has a direct relation to necessity of oxygen supply in COVID-19 patients [36].

## 5. TMPRSS2 in Other Human Diseases

### 5.1. SARS

Similar to SARS-CoV-2, SARS-CoV docks in the ACE2 receptor of host cell membrane, using its S1 subunit for binding and the S2 subunit for fusion [61]. Few proteases such as cathepsin L, elastase, trypsin, factor Xa, thermolysin, and plasmin were thought to have roles in activating the S2 subunit of the viral spike protein [62–65]. Matsuyama et al. first presented data on the correlation between TMPRSS2 expressions in the lung with SARS-CoV, indicating that the protease may have significant contribution in activating the virus spike protein to induce its fusion with cell membrane [66]. Their study further suggested that opposite spatial orientation of the proteins is necessary for membrane fusion and TMPRSS2 can only act on spike proteins already attached to the receptor, as the cleavage site in the spike protein is only exposed after the receptor docking of the protein [66]. Later, a series of studies strengthened this prediction that TMPRSS2 plays a more significant role in comparison with the other proteases in SARS-CoV infection [61, 62]. Glowacka et al. showed that, aside from assisting the fusion of the virus cells, TMPRSS2 protein reduces the ability of the neutralizing antibodies to recognize the virus [67].

### 5.2. Influenza

The fusion of influenza virus and host cell membrane is facilitated by the binding of the viral surface glycoprotein hemagglutinin (HA) with the receptor [68]. The host proteases play an important role in processing of the hemagglutinin precursor into HA1 and HA2 subunits, a step essential for the fusion process [69]. The protease has been found to play this role for different subtypes of influenza A and influenza B virus, and several studies carried out in knockout and knockdown mice have proved that absence of TMPRSS2 expression leads to resistance against the influenza virus [68–73]. Limburg et al. suggested that the potential inhibition of TMPRSS2 can be an effective therapeutic option against human influenza as TMPRSS2 is essential for the activation and multiplication of the virus [69]. Further studies found that polymorphism in the TMPRSS2 protein can affect the severity of influenza in humans [74, 75].

### 5.3. MERS

The spike protein (S) of MERS-CoV, the Middle East respiratory syndrome virus, attaches itself with the cell surface receptor of host cell. Instead of ACE2, the MERS virus uses the dipeptidyl peptidase 4 (DPP4) receptor, which is abundantly present in epithelial and endothelial tissues for binding [76]. This process is carried out by the spike protein (S) anchored in the virus cell membrane. Matsuyama et al. observed that TMPRSS2 along with cathepsin L is responsible for the priming of MERS virus spike protein and is, therefore, responsible for the successful cellular entry of the virus [77]. As TMPRSS2 has been found to be expressed in higher level in the respiratory epithelium, in comparison with cathepsin L, Kleine-Weber assumed that TMPRSS2 plays the primary role among these two proteases in the S protein priming, which ensures the viral fusion with the host cells [78].

### 5.4. Metapneumovirus Infection

Aside from the respiratory viral diseases discussed above, TMPRSS2 has also been found to be involved in human metapneumovirus (HMPV) infection, which is responsible for bronchiolitis and pneumonia [79]. Shirogane et al. reported that TMPRSS2 cleaves the HMPV fusion protein and actively assists in viral multiplication in host cell [79].

### 5.5. Inhibitors of TMPRSS2

Host protease inhibition has been under consideration as a low risk option for inhibiting some viral infections in recent years [80]. The role of TMPRSS2 in the pathogenesis of cancer and various infectious diseases of viral origin has led to several attempts to inhibit its activity as a way of preventing and treating disease progression. Soon after the emergence of COVID-19, hundreds of clinical and preclinical trials started to investigate the effectiveness of drugs on multiple targets and protease inhibition is one of the popular approaches.

Camostat and nafamostat are two protease inhibitors which have shown successful inhibitory effect on TMPRSS2 in both *in vitro* and *in vivo* studies [81]. Hoffmann et al. suggested the potential application of camostat mesylate for TMPRSS2 inhibition [7]. Camostat is approved in Japan for chronic pancreatitis and had been investigated for other therapeutic options including cancer and dyspepsia [82]. A previous study showed that it can interfere with influenza virus replication by inhibiting TMPRSS2 and other serine proteases [83]. Another similar drug, nafamostat mesylate, is approved in Japan for the treatment of acute pancreatitis [82]. It had shown successful TMPRSS2 inhibition in MERS-CoV infection [82, 84]. As the SARS-CoV-2 spike protein had similarity with the S protein of the formerly mentioned coronavirus, this drug has considerable potential against the SARS-CoV-2 spike protein and is under investigation for this purpose [82]. Nafamostat mesylate has already shown success in inhibiting the protease in simian Vero E6 cells [82, 85]. There are sufficient data on the safety of this molecule at a dose level of 240 mg for 5 days [82, 86]. The widely applied cough suppressant bromhexine hydrochloride is another compound with considerable potential for TMPRSS2 inhibition based on large-scale screening data [87, 88]. Based on the evidence that TMPRSS2 has a role to play in cancer metastasis, bromhexine was administered systemically to cancer patients to observe its effectiveness in reducing the metastasis [25, 88]. The study outcome was positive in favour of bromhexine use with no potential systemic side effects [25, 88]. Aprotinin is another serine protease inhibitor known to be effective in inhibiting TMPRSS2 and few other proteases in cell culture studies [89]. Numerous *in silico* studies were undertaken in the recent months after the emergence of COVID-19 to investigate new molecules, few of which are studying protease inhibitors for TMPRSS2. These include screening of commercially available compounds for their possible inhibitory effect [90]. Screening of natural product database using computer-aided strategies is also under consideration [91]. It was found that steroids help in upregulation of TMPRSS2 [52]. A study found that treatment with particular estrogens can decrease the severity of the disease through suppressing the expression of the protease hormone [92]. In the abovementioned theoretical basis, it is hypothesized that antiandrogen therapy in males could be effective against the viral disease [93]. Studies conducted so far have not shown any significant role of TMPRSS2 in biological processes leading to the assumption that other serine proteases might compensate for its absence [67]. A study found that the gene is dispensable for normal development, growth, and organ function in knockout mouse model [94]. These findings increase the possibility that inhibiting TMPRSS2 expression will not have any significant side effect. Contrarily, furin, the other protease involved in the proteolytic processing of the spike protein, is known to be involved in several biological processes [13]. This makes TMPRSS2 a more feasible target of choice for drug development. Still there is a possibility that some biological roles of the protease have not been fully elucidated yet. So, before targeting the protease activity of TMPRSS2, further studies on its role in physiological processes are essential.

## 6. Conclusion

The expression pattern and the role of TMPRSS2 in several infectious diseases render significant level of safety in assuming that the protease has a major role in cellular entry of SARS-CoV-2 in human cells. This idea has given new impetus to the protease inhibitor development projects. Studies conducted so far have proved the safety profile of few protease inhibitors, some of which are discussed in this article. COVID-19 clinical trials using protease inhibitors have already started to enroll patients. Concurrent studies on the physiological role of TMPRSS2 in humans will produce better evidence in favour of the application of protease inhibitors for COVID-19. If molecules with satisfactory safety and efficacy profile can be developed, those will have high potential of reducing the infection rate and severity of the disease.

## Data Availability

No data were used to support this study.
